# The Biobank Portal for Partners Personalized Medicine: A Query Tool for Working with Consented Biobank Samples, Genotypes, and Phenotypes Using i2b2

**DOI:** 10.3390/jpm6010011

**Published:** 2016-02-26

**Authors:** Vivian S. Gainer, Andrew Cagan, Victor M. Castro, Stacey Duey, Bhaswati Ghosh, Alyssa P. Goodson, Sergey Goryachev, Reeta Metta, Taowei David Wang, Nich Wattanasin, Shawn N. Murphy

**Affiliations:** 1 Partners HealthCare, One Constitution Center, Boston, MA 02129, USA; acagan@partners.org (A.C.); vcastro@partners.org (V.M.C.); sduey@partners.org (S.D.); bghosh3@partners.org (B.G.); agoodson@partners.org (A.P.G.); sgoryachev@partners.org (S.G.); rmetta@partners.org (R.M.); tdwang@partners.org (T.D.W.); nwattanasin@partners.org (N.W.); snmurphy@partners.org (S.N.M.); 2 Department of Neurology, Massachusetts General Hospital, Boston, MA 02114, USA

**Keywords:** Biobank IT, personalized medicine IT, precision medicine IT, Biobank software, Biobank information technology, phenotype

## Abstract

We have designed a Biobank Portal that lets researchers request Biobank samples and genotypic data, query associated electronic health records, and design and download datasets containing de-identified attributes about consented Biobank subjects. This do-it-yourself functionality puts a wide variety and volume of data at the fingertips of investigators, allowing them to create custom datasets for their clinical and genomic research from complex phenotypic data and quickly obtain corresponding samples and genomic data. The Biobank Portal is built upon the i2b2 infrastructure [1] and uses an open-source web client that is available to faculty members and other investigators behind an institutional firewall. Built-in privacy measures [2] ensure that the data in the Portal are utilized only according to the processes to which the patients have given consent.

## 1. Introduction

The Partners Biobank is a collection of plasma, serum, and DNA samples of consented subjects linked to their electronic health records (EHR) with the aim of fostering clinical and genomic discovery. For the biospecimen-linked EHR to be maximally useful for research, investigators must be able to access, query, download, and analyze it while following the regulations set out by the Institutional Review Board. Data issues such as quality, timeliness, storage, acquisition, distribution, security, and interpretation must be addressed in the implementation. The Partners Biobank Portal is an open-source application based on the i2b2 infrastructure (https://www.i2b2.org/) [1]. It was created to enable Partners researchers to query and download data about Biobank subjects and make requests for samples and genomic data, while addressing these issues. The Biobank Portal effectively links dispersed information about Biobank subjects, including:

- Available sample types,
- Electronic health record data,
- Genotypic results,
- Patient-completed health surveys, and
- Statistically-computed phenotypes.

Due to the dynamic structure of the i2b2 software and the flexibility of the underlying database, as new subjects are consented to the Biobank, and as more and different data on the subjects become available from different sources in the enterprise, they are easily added and made available to investigators in the Biobank Portal. This gives researchers access to comprehensive and timely data in a secure and useable interface.

The aim of this manuscript is to describe methods and tools developed to help investigators work with Biobank data and samples using the i2b2 framework in the Partners Biobank Portal. 

## 2. Results

A goal of the Biobank Portal is to integrate diverse types of data about Biobank subjects in a secure database in order to make it possible to address a range of clinical research questions while maintaining patient confidentiality. A central SQL Server database contains data from different sources, such as the Electronic Health Record and patient-reported surveys and the Biobank itself. Also included is derived data in the form of validated phenotypes and a Charlson co-morbidity index for finding healthy subjects to use as controls. We created a mechanism to perform weekly updates to the database. These updates add new subjects, all data related to the new subjects, and all new data related to existing subjects, so that researchers have access to up-to-date information about patients and their samples.

Several data security measures have been employed. All data in the Biobank Portal are available as a coded limited dataset (LDS) as defined by HIPAA’s Privacy Rule [3]. “Coded” means the data conform to the definition of an LDS but, unlike a pure LDS, the coded patient data are linked to identifiers that allow them to be updated. The Biobank Portal requires users to sign an electronic data use agreement (DUA) in compliance with both the Privacy Act and HIPAA upon registration. The Biobank Portal takes an additional step to obfuscate the LDS and, thus, provide further de-identification. All dates are shifted and zip codes are truncated to 3 digits.

**Figure 1:**
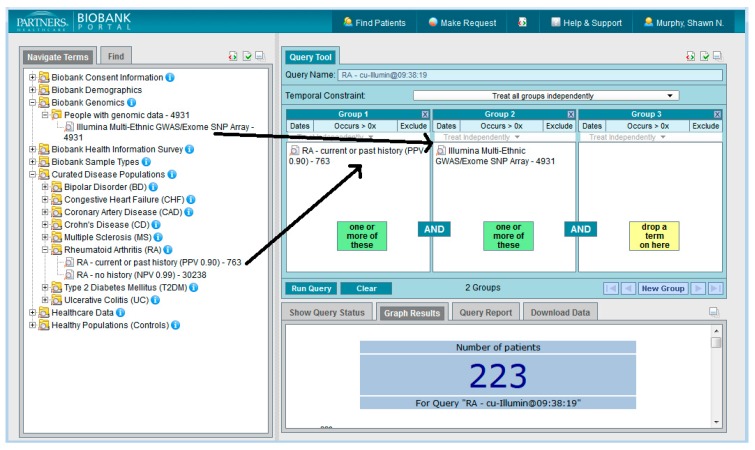
The Biobank Portal.

A web client application was built using i2b2 software [1,4,5]. This application, the Biobank Portal, provides the ability for investigators to query all of the assembled data types to come up with patient sets, or cohorts, for further study. Researchers must register to use the tool with a valid institutional logon and may only access the tool from within the Partners firewall. The interface is designed to be easy and intuitive to use with very little training [6]. Figure 1 shows the Biobank Portal UI in which a query has been constructed to look for all subjects who have rheumatoid arthritis according to a validated phenotype with 90% PPV and have genotypic data available.

We have also developed extensive user-facing help in the form of an interactive tutorial, which guides the user through the workings of the Portal, and a wiki, which includes specific information and references about our methods and the provenance of the data, as well as how to contact us for assistance.

The Portal can be used not only to create queries, but to create spreadsheets of LDS data and to request samples and genomic data. Investigators can run a query and then create a spreadsheet for selected variables for the subjects returned by the query. For example, a researcher can run the query above (rheumatoid arthritis and genotyped) and may then want to see how many anti-TNF medications the resulting patients have taken. Using the Portal, they can select all the anti-TNF medications of interest into the spreadsheet creation form, click a button, and download the data in an Excel file. They can work with the data and select the subjects for whom they wish to request either samples or genetic data and then make the request in the Portal. Rules from previous work can be formulated into an i2b2 query and these can be published in the Query Tool workplace folders to be shared with other users

The robust and flexible infrastructure of i2b2, which uses modular components, allows for data growth and the easy addition of new functionality. It also provides built-in auditing measures that allow us to keep track of work that is being done, such as all queries that have been run and all requests that have been made.

**Figure 2:**
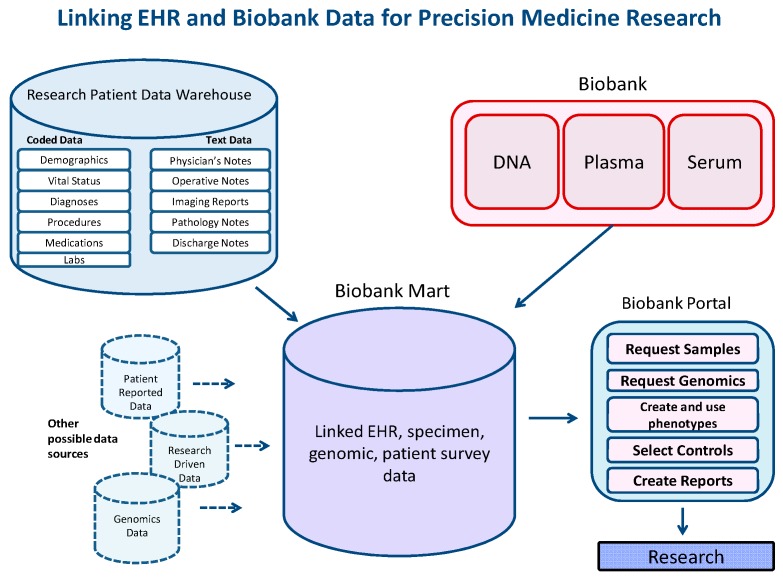
Linking EHR and Biobank data for precision medicine research.

Currently, over 35,000 people have volunteered to participate in research via the Partners Biobank. Figure 2 shows the diverse data types amassed in the SQL Server Biobank Portal database for these consented Biobank patients. The data include bio-specimens (DNA, plasma, and serum), the results of genotyping nearly 2 million genomic variants (on a subset of the cohort), behavioral data in the form of a patient-reported survey, data from the Electronic Health Record, and research-related derived data (Charlson index, validated phenotypes). All of these data are made available to qualified researchers in the Biobank Portal web tool. Regarding performance, an average of a set of queries ranging in complexity from only one item in one panel to five panels with up to three elements in each (a combination of terms “and-ed” and “or-ed” together) and returning between 150 and 25,000 patients, takes between 3 and 20 s to return aggregate totals.

The EHR can be a major source of data for clinical and translational research; however, the quality of the data is highly variable [7,8,9,10]. Codes that are used to designate patient diseases are often used by clinicians to designate possible rather than definitive diagnoses. A focus of the Biobank Portal is the creation and implementation of rigorously-defined phenotypes to provide a more accurate picture of clinical conditions. The phenotype algorithms result in cohorts of subjects who we can say have a particular disease with a high positive predictive value, which can then be used by investigators for study with full confidence that the patients have the disease, or at least an understanding of the accuracy to which they know the patient has the disease. Otherwise, the investigator would need to take an enormous amount of time to perform chart reviews to identify individual subjects [11,12]. The phenotypes, which typically identify a large set of subjects, can then be helpful for achieving the power required by genetic studies to detect risk alleles associated with disease.

A set of phenotypes was developed within the Biobank population to identify which patients had particular diseases with high statistical probability. We found that looking at ICD-9 codes alone was usually not sufficient for saying someone had a particular disease. Structured (coded) data are primarily used for billing and administrative purposes, so these data are inaccurate for cohort identification and can inflate the number of subjects classified as having the phenotype (false positives). Table 1 shows the number of patients who have ICD-9 codes for a set of diseases *vs.* the number who were determined to have the diseases based on the phenotype algorithms. The ICD-9 column is a higher number, but many of these represent false positives.

To add continuity and clarity to the coded data, we included textual reports in our phenotype development, both for chart review for test sets and validation, and as an added source of information about the disease in question [12]. There are about five million clinical narratives associated with the Biobank patients, which record the details of patient-provider interactions, such as reasons for visits, diagnostic tests given, medication alterations, and suspected or confirmed disease. We used Natural Language Processing (NLP) to extract relevant concepts from the text reports. Chart reviews of narrative data were done to create gold standard training and validation sets for each algorithm [12].

For each phenotype, we created an analysis file of concepts that included potential positive and negative predictors of the disease. The variables were made up of both coded terms and terms extracted from the narrative data. We then used the adaptive LASSO penalized logistic regression method to identify predictive variables and their relative weights for the algorithm [13]. More important than the accuracy of any variable alone was how the variables together in the algorithm could predict the phenotypes. For the final classification algorithm, we applied a logistic regression model that assigned each subject a probability of having a phenotype based on their values for each term. We set a threshold and classified patients as having or not having a phenotype based on whether their probability was above or below the threshold. The ability to change the threshold allows the investigator to make changes to suit the problem at hand, unlike in a rules-based approach. For example, if more patients are needed for a study, using a lower specificity threshold can improve the power of the algorithm.

**Figure 3:**
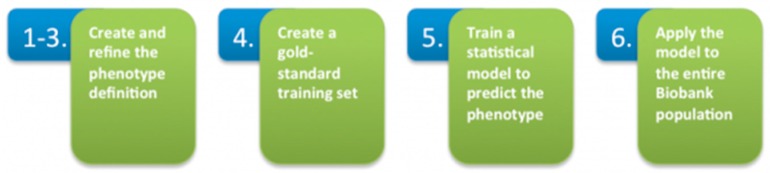
High-throughput phenotyping.

This high-throughput phenotyping process, summarized in Figure 3, produced robust phenotype algorithms that were evaluated using metrics such as sensitivity, the proportion of true positives correctly identified as such, and positive predictive value (PPV), the proportion of individuals classified as cases by the algorithm. As the Biobank population grows, the model can be rapidly redeployed to refresh potential cohorts using the same standardized phenotype definition.

(1) Create an initial phenotype definition using diagnosis codes.
(2) Broaden the definition by determining the most up-to-date features (co-morbidities, symptoms, medications) that create a more accurate profile of the phenotype when combined with ICD-9 codes.
(3) Narrow and refine the definition by determining the features that occur most often in the Biobank data. Extract, code, and rank features contained in clinical narratives with Natural Language Processing (NLP).
(4) Create a gold-standard patient set for training the method. Query coded EMR data for the set of patients having at least one diagnosis code for the phenotype. Apply a statistical sampling algorithm to select a random subset of those patients for full chart review. A clinical expert performs a full chart review to classify the patients as positive or negative for the phenotype.
(5) Train a statistical model that incorporates all features in the definition to predict the presence or absence of the phenotype against the gold-standard patient set.
(6) Apply the trained model to the entire Biobank population.

## 3. Discussion

Other phenotype efforts also aim to model diseases using secondary EHR data, notably eMERGE, which uses clinical expertise to create rules-based disease definitions that are applied over diverse EHRs at different sites [14]. Our approach is probabilistic and requires the machine-learning described here. Both methods take time to develop, due to the idiosyncrasies of structured and narrative EHR data. Patient notes are a rich source of information, but they are recorded in ways that, though efficient and convenient to practitioners, are less so for analysis. They usually do not conform to standards, often contain misspellings and alternate ways of expressing concepts, and there may be hundreds of notes for a single patient alone, all of which make the process of extracting meaningful information difficult. We used NLP to extract relevant concepts from the text reports. While incorporating NLP improved the performance of the algorithms, it was also time-consuming, as it required an NLP expert to develop algorithms for finding the correct information for every algorithm. Often, a clinical expert also had to be consulted to make sure the correct terms were being used. Chart reviews of narrative data, essential for creating a gold standard and validation set for each algorithm, proved to be the most rate-limiting step due to the volume and complexity of the notes, and the manual process of reviewing charts. We are currently working on ways to streamline some of the processes involved. Recently, a method for automated feature extraction from knowledge sources was described to help reduce some of the manual effort involved in creating phenotypes [15].

We have investigated how dependent the algorithms are to the specific institution at which they are developed. A rheumatoid arthritis phenotype algorithm developed at Partners Healthcare was applied to datasets from Northwestern and Vanderbilt Universities which used different EHR systems, different NLP systems, and different target NLP vocabularies [16]. Rapid identification of case populations was shown at each site with little retraining. We hope to further explore issues of reproducibility as our work progresses.

Another challenge is educating the research community about the statistical phenotyping techniques we have developed. Genetics researchers, as a rule, are not familiar with EHR data and the complexity within it, so it is often difficult for them to appreciate the usefulness of the algorithms without explanation. Therefore outreach and education must be developed to disseminate this information. On the other hand, we, the developers of the software tools, are also learning from researchers what their needs are regarding both phenotypic and genotypic data. The give and take between the end-users and the developers and analysts has been, and continues to be, a vital part of the development of these tools.

There are many biobanking efforts in progress around the world that link consented samples with EHR and other data for personalized and translational medicine [17,18,19]. Most of these rely on a “concierge service” to return data and samples to investigators. They often require that an online form is filled out to specify the criteria required for selecting samples and data, which is then gathered by an analyst and eventually returned to the investigator. If the analyst does not fully understand the requirements in the way the researcher intended, this process can become iterative and take a lot of time. The Biobank Portal employs a self-serve approach to alleviate this bottleneck between the researcher and the analyst, allowing the researcher to perform independent feasibility analyses and to directly control their data design and specifications. For large institutions with many researchers requesting analytics on Biobank data, this provides immediate service and the ability to explore and better understand the available data.

## 4. Materials and Methods

### 4.1. Rules for Accessing the Biobank Portal

New registrants must sign an electronic data use agreement (DUA) to ensure that the data remain protected against unauthorized disclosure as outlined in the consent agreed to by each Biobank participant. The Biobank Portal can only be accessed from within the Partners firewall. Registered users log in using their Partners NT logons and passwords. Once users register and log in, they can use the Biobank Portal to create queries, download data, and make requests for samples and genotyping data as authorized by the patient consent.

### 4.2. Data in the Biobank Portal

Data in the Biobank Portal are available as a coded limited data set (LDS) as defined by HIPAA’s Privacy Rule. Limited datasets may be used for research, public health, or health care operations. They are not directly identifiable, but may contain the following protected health information (PHI): town, city, state or zip code, and elements of dates related to a person: years, birth dates, admission dates, discharge dates, and dates of death. Disclosing the PHI in a LDS requires that the covered entity enter into a data use agreement with the recipient who must agree to use the data for limited purposes. The Biobank requires users to sign an electronic DUA to this effect upon registration.

The Biobank Portal takes an additional step to obfuscate the LDS and thus provide further de-identification. All dates are shifted and zip codes are truncated to three digits.

Data in the Portal are stored in a SQL Server database in the star schema format defined by i2b2.

The database is updated with new subjects who have been consented to the Biobank and their associated data every week and this data are made available to users in the Biobank Portal Query Tool.

The user interface of the Portal is in i2b2 format, with vocabulary terms for all the data types described below located in the upper left hand side in the Navigate Terms panel and the Query Tool panels on the right. Users can expand the folders of data in the Navigate Terms panel, select query criteria, and drag them into the query panels to construct queries. The Query Tool panels follow Boolean logic, with terms grouped in the same panel being treated as logical “OR” and terms in different panels treated as “AND”.

### 4.3. Data Types in the Biobank Portal

**Figure 4:**
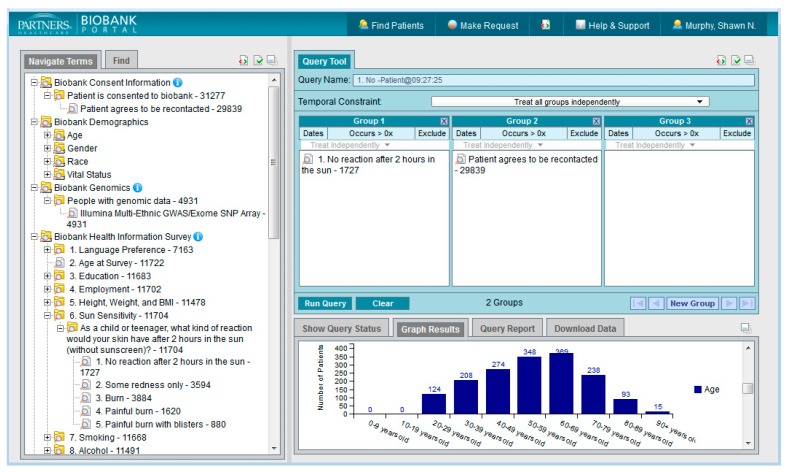
The Biobank Portal with expanded ontology folders.

Figure 4 shows the Biobank Portal Query Tool. In the upper left hand side in the Navigate Terms box are the ontology types that may be expanded and used as query items. The i2b2 data structure was designed so that new “ontologies” can be easily added for specific custom uses as long as the documented format for i2b2 metadata is followed. The format is hierarchical and the display of the vocabulary items in the UI mimics the hierarchy described in the underlying i2b2 SQL Server table. The figure shows two folders that have been opened to their constituent folders, “Biobank Consent Information” and “Healthcare Data”. The data types described below contain many folders of information in a hierarchy that can be drilled into to find needed terms. Terms and codes may also be searched for using the Find Terms tab. The structure of the query tool is flexible and allows for many different data types to be added. Some of the current data types available in the Portal are listed below.

#### 4.3.1. Biobank Consent Information

All Biobank subjects have provided their consent to join the Partners Biobank, which includes agreeing to provide a blood sample linked to the electronic medical record. Subjects also agree to be re-contacted by the Partners Biobank staff as needed. These attributes may be queried alone or in combination with other data types in the Biobank Portal when making a sample request to determine how many patients have consented to join the Partners Biobank and how many consented patients have agreed to be re-contacted by the Biobank staff.

#### 4.3.2. Biobank Demographics

The Biobank Demographics folder in the Navigate Terms panel contains subfolders for age, gender, race, and vital status. Users can expand these folders select the underlying terms of interest to drag into the Query Tool panels when constructing queries to specify which variables they are interested in (e.g., white females between the ages of 5 and 20 who are alive.)

#### 4.3.3. Biobank Genomics

25,000 of the Biobank’s subjects are in the process of being genotyped and the data made available to the research community. The Biobank Genomics attribute can be used in a query to find out how many of the subjects’ DNA has been genotyped.

#### 4.3.4. Health Information Survey

The Health Information Survey in the Biobank Portal provides patient-reported lifestyle, environment and family history information. All Biobank subjects are asked to fill out an online health information survey on a comprehensive list of health-related topics, including variables about their education, employment, sun exposure, smoking, alcohol and sleep habits, and family history of certain diseases. These attributes may be queried alone or in combination with other data types in the Portal to create patient sets for specific study. For an epidemiological study on the effects of shift work on hormonal levels, for example, one researcher queried the sleep data from the survey.

#### 4.3.5. Biobank Sample Types

Users are advised that, when making a sample request, they run a query to determine whether the subjects identified in the Biobank Portal have the desired sample type(s). This folder contains the sample types available for each consented patient.

#### 4.3.6. Curated Disease Populations (Validated Phenotypes)

Curated Disease Populations in the Biobank Portal are calculated disease definitions, or phenotypes, for the Biobank subjects. These phenotypes were developed by the Biobank Portal team (as a continuation of work that was done in the i2b2 Driving Biology Projects) using both structured and unstructured electronic health record data and clinical, computational and statistical methods. Each phenotype was initially defined by ICD-9 diagnosis codes. The most up-to-date co-morbidities, symptoms, and/or medications (features) were extracted from online medical literature and knowledge bases and used to broaden the phenotype definition. The feature set was narrowed to those that are most relevant to the Biobank population through Natural Language Processing (NLP) of all clinical narratives. A gold-standard training set of patients was determined to have or not have the phenotype by expert review of clinical narratives. The gold-standard set was used to train the model to accurately predict the phenotype based on the refined definition. The method was applied to the entire Biobank population.

The resulting phenotypes can be used to identify Biobank subjects who may, with a certain positive predictive value, have these conditions. As more phenotype definitions are developed, they can easily be added to the library in the Biobank Portal.

#### 4.3.7. Healthcare Data

The Healthcare Data folder in the Portal contains coded data from the EHR, including ICD, LOINC, CPT, HCSPCS, and local codes for diagnoses, procedures, lab tests, medications, and details about patient visits. The data types are arranged by subject and coding system hierarchies, where they exist. The Healthcare Data types are the most used variables in the Portal. Investigators use them to create queries that to define patient sets based on patient problems and treatments.

#### 4.3.8. Healthy Populations (Controls)

The Healthy Population category in the Biobank Portal is designed to help select relatively healthy controls from the Biobank population. The calculations are based on the Charlson Age-Comorbidity Index that combines the presence and severity of comorbidities with age to predict the 10-year survival probability for Biobank Portal subjects [20,21].

To calculate the index, ICD-9 diagnostic codes are grouped into 17 weighted comorbidity categories (Table 2) [22]. The sum of the weights for each comorbidity present is added to an age score (Table 3) to calculate the index for each subject (Table 4). The index is translated to a single prognostic value.

(Sum of the weights for each comorbidity category present + Age Score) = *X*

*e*^(0.9*X)^ = *Y*

0.983*^Y^* = *Z* (10-year survival probability)

The Charlson Index may be used to assess the severity of illness for individuals or a population of subjects; however, it does not provide a population completely free of disease or chronic conditions.

## 5. Conclusion

We describe using the i2b2 framework to build the Biobank Portal for Partners Personalized Medicine. The Portal allows researchers to create and run complex queries of EHR and other data, download LDS for further analysis and make requests for genomic data and samples. The goal of the Portal is be a self-service tool for all Biobank-related functions and to inform further development that will assist with using genomic information, EHR data and bio-samples in clinical research.

## Figures and Tables

**Table 1:** Number of patients estimated to have a disease based on 1 or more ICD-9 code alone *vs.* the phenotyping algorithm.

	1 or More ICD-9 Alone	Phenotype Algorithm
Bipolar Disorder	405	125
Coronary Artery Disease	3611	3287
Crohn’s Disease	657	562
Congestive Heart Failure	1130	487
Multiple Sclerosis	212	160
Rheumatoid Arthritis	787	687
Type-II Diabetes Mellitus	3331	2845
Ulcerative Colitis	518	400

**Table 2:** Weighted comorbidity categories.

Category	Weight
Myocardial infarction	1
Congestive heart failure	1
Peripheral vascular disease	1
Cerebrovascular disease	1
Dementia	1
Rheumatologic disease	1
Chronic pulmonary disease	1
Peptic ulcer disease	1
Mild liver disease	1
Diabetes (mild to moderate)	1
Diabetes with chronic complications	2
Hemiplegia or paraplegia	2
Renal disease	2
Any malignancy, including lymphoma and leukemia	2
Moderate or severe liver disease	3
Metastatic solid tumor	6
AIDS	6

**Table 3:** Age-based score.

Age	Points
<50	0
50–59	1
60–69	2
70–79	3
80–89	4
90+	5

**Table 4:** The Charlson Index.

Charlson Index	10-Year Survival
0	98.30%
1	95.87%
2	90.15%
3	77.48%
4	53.39%
5	21.36%
6	2.25%
≥7	≤0.009%
